# Bacterial Pneumonia and Pandemic Influenza Planning

**DOI:** 10.3201/eid1408.070751

**Published:** 2008-08

**Authors:** Ravindra K. Gupta, Robert George, Jonathan S. Nguyen-Van-Tam

**Affiliations:** * John Radcliffe Hospital, Oxford, UK; † Health Protection Agency, London, UK; ‡ University of Nottingham, Nottingham, UK

**Keywords:** Pandemic influenza, secondary bacterial pneumonia, antibiotic stockpiles, pneumococcal vaccination, emergency planning, perspective

## Abstract

Pandemic influenza planning is well under way across the globe. Antiviral drugs and vaccines have dominated the therapeutic agenda. Far less work has been conducted on stockpiling and planning for deployment of antimicrobial drugs against secondary bacterial pneumonia, a cause of substantial illness and death in previous pandemics and epidemics. In the event of a pandemic, effective antimicrobial drug measures are expected to substantially benefit public health. We address issues regarding use of antimicrobial drugs as stocks of individual agents are diminished and the role of resistance surveillance in informing such policy. Furthermore, vaccination with polysaccharide and conjugate pneumococcal vaccines is considered as part of a pandemic strategy. Most illness and death from influenza are likely to occur in developing countries, where neuraminidase inhibitors and vaccines may be neither affordable nor available; thus, compared with industrialized countries, the benefits of treating bacterial complications in developing countries may be substantially greater.

## Abstract 2

Prevention and treatment of secondary bacterial complications are important but neglected areas of planning.

The threat of a pandemic has been raised by the recent emergence of avian influenza virus (H5N1) in Southeast Asia. If an influenza pandemic of the same magnitude and severity as the one in 1918–19 were to occur in the present day, worldwide an estimated 51–81 million persons would die (*1*).

To date, antiviral drugs, principally the neuraminidase inhibitors, and vaccines have dominated the pharmaceutical countermeasures agenda in terms of research and development, stockpiling, and planning for mass deployment. However, the global supply of neuraminidase inhibitors is likely to be limited, and an immunogenic vaccine matched specifically to the pandemic strain would take at least 4–6 months to produce. Effective public health measures are predicted to slow, rather than halt, the spread of infection. Large numbers of influenza cases are therefore likely to occur when a pandemic strain emerges.

Evidence from laboratory, clinical, and epidemiologic studies suggests that bacterial co-infection contributes substantially to the illness and death that occurs in pandemic and seasonal influenza. We consider bacterial co-infection in the context of current preparedness activities and guidelines regarding antimicrobial drug stockpiling and deployment, including reference to existing quinolone stockpiles held by a number of countries. We also discuss the potential role of vaccination against *Streptococcus pneumoniae* in the context of pandemic influenza.

## Bacterial Pneumonia and Pandemic Influenza

Ecologic studies have demonstrated temporal relationships between influenza activity and bacterial pneumonia. This association was perhaps most strikingly emphasized by the 20th-century pandemics, which have been comprehensively reviewed by Brundage (*2*). Substantial laboratory evidence for synergism between influenza A and bacterial agents has been reviewed by McCullers (*3*).

### Bacterial Pneumonia and Seasonal Influenza

Pandemics are relatively rare; therefore, more data are available about bacterial infections associated with seasonal than pandemic influenza A strains. Secondary bacterial pneumonia is a common cause of death in persons with seasonal influenza; co-infections have been found with ≈25% of all influenza-related deaths (*4*,*5*). Pathogen-specific data are summarized below.

### Streptococcus pneumoniae

Of laboratory-confirmed cases of community-acquired pneumonia, ≈30% involve bacterial–viral co-infection (*6*–*8*). *S. pneumonia* is the most common cause of community-acquired pneumonia and bacterial co-infection with influenza A (*9*–*12*). Invasive pneumococcal disease is a term used when the organism is isolated from a typically sterile site, such as blood or pleural fluid. This definition therefore underestimates pneumococcal pneumonia where isolation of the organism is not possible (*13*). Notwithstanding, a number of studies have documented the temporal association between influenza and invasive pneumococcal disease, which suggests synergism. Grabowska et al. (*14*) recently used 2 epidemiologic methods based on Swedish surveillance data to estimate the excess cases of invasive pneumococcal pneumonia associated with seasonal influenza at 12%–30%.

HIV-infected children have a 40× greater risk than HIV-noninfected children for invasive pneumococcal disease and account for most cases of invasive pneumococcal disease in certain sub-Saharan African countries (*13*,*15*). HIV-infected children and adults would likely be more severely affected by an influenza pandemic.

### *Staphylococcus aureus* (Methicillin Sensitive and Methicillin Resistant)

A retrospective study of influenza-related childhood deaths in the United States in the 2003–04 season found *S. aureus* to be the most common bacterial agent, accounting for 46% of isolates, >50% of which were methicillin-resistant strains (*5*). Surveillance for severe influenza-related *S. aureus* community-acquired pneumonia in the United States during the 2003-04 season recorded 17 cases (88% methicillin-resistant *S. aureus* [MRSA]) and 5 deaths (4 with MRSA) and a median age of 21 years (*16*); laboratory evidence of influenza infection was available for ≈75%. More recently, 10 cases of severe community-acquired MRSA pneumonia in children (6 of whom died) from 2 southern states in a 2-month period were reported (*17*). For 30% of those patients, MRSA was recovered from sputum only, and 4 had a documented recent history of MRSA skin infection in themselves or in a close contact. Preceding staphylococcal skin disease in persons with staphylococcal pneumonia was described by Goslings et al. (*18*) during the 1957–58 pandemic. In the context of emerging community-acquired MRSA skin infection in persons without traditional risk factors, this association has substantial implications for possible emergence of MRSA pneumonia in a future pandemic (*19*)*.*

### Other Pathogens

A recent study from New Zealand (*7*) that aimed to characterize viral causes of community-acquired pneumonia reported viral–bacterial co-infection in 45 (15%) of 304 hospitalized patients. *S. pneumoniae (*67%) and *Haemophilus influenzae* (11%) were the 2 pathogens most commonly associated with influenza A infection; atypical microbes (*Chlamydia pneumoniae, Mycoplasma pneumoniae,* and *Legionella pneumophila*) were also well represented (22%). These figures are generally consistent with other published data; group A streptococci are a rare but serious cause of community-acquired pneumonia (*20*) and have been associated with fatal cases of influenza (*5*).

### Stockpiling and Strategic Use of Antimicrobial Drugs

In most modern healthcare systems, which increasingly emphasize just-in-time supply chains, shortages of antimicrobial drugs may occur rapidly unless more are stockpiled. These shortages would limit the treatment of secondary bacterial infections in the middle and the later stages of a pandemic. For this reason a range of antimicrobial drug options have been suggested, taking into account the likely limitations of availability in diagnostics for community-acquired pneumonia and the fact that, because of the sheer number of patients, therapy is likely to be empirical. Clinical management guidelines for pandemic influenza have recommended amoxicillin + clavulanate or doxycycline (*21*); third-generation cephalosporins or respiratory fluoroquinolones (*22*); and second-generation cephalosporins, macrolides, doxycycline, or co-trimoxazole (*23*) as first-line empirical therapies for community-acquired pneumonia associated with pandemic influenza. Dependent on the extent of any stockpile, shortages of these preferred agents might occur first during a pandemic.

In the United States, the emergence of community-acquired MRSA has prompted revision to include vancomycin and other agents as empirical therapy for severe cases (*21*,*22*). The demand created by empirical use of vancomycin in such cases, the limited number of alternative agents, and the limited global production capacity of this drug are likely to lead to its shortage. Other treatment possibilities include linezolid, quinopristin/dalfopristin, and tigecycline.

Fortunately, in the United Kingdom most MRSA isolates are sensitive to doxycycline (95% of respiratory isolates; Health Protection Agency [HPA], unpub. data) and rifampin (97%; HPA, unpub. data); fewer are sensitive to trimethoprim (72%). Less severe MRSA infections treated with these widely available and inexpensive drugs would be expected to respond. Rifampin and cotrimoxazole are widely produced in developing countries, where the prevalence of tuberculosis and HIV infection are high.

### Real-time Surveillance of Pathogen Resistance

After a country has committed to acquiring a stockpile of antimicrobial drugs, several important practical and logistic issues arise. The first is deciding on the range of antimicrobial drugs to be stockpiled. After the World Health Organization declares a global pandemic phase 5 alert, antimicrobial drug supplies will be quickly depleted as countries scour the global market to build up stocks. The choice of available agents may be limited by this stage; therefore, procuring in advance is sensible, although this involves predicting which bacterial agents will be of greatest importance. The UK HPA has developed a program of real-time surveillance of antimicrobial susceptibility for the 3 most likely influenza-related bacterial pneumonia pathogens: *S. pneumoniae*, *H. influenzae,* and *S. aureus.* Contemporaneous data are available for each pathogen, enabling recommendation of antimicrobial drugs on the basis of the proportion of respiratory tract isolates likely to be susceptible at a particular point in time. Such real-time data may be useful for guiding the evolution of pandemic antimicrobial drug treatment policy in order to optimize the use of scarce antimicrobial drugs by drawing on a range of different agents according to national stock availability at the time. The surveillance program may also provide early warning of likely clinical failures caused by emerging resistance.

### Size, Storage, and Turnover of Stockpiles

Decisions about pandemic stockpiles, procurements, and size depend primarily on financial considerations. Decision-makers must bear in the mind the need not only to purchase the initial stockpile but also to maintain it, perhaps for a sustained period. In most circumstances, stockpiles of vaccines for influenza virus subtype H5N1 and neuraminidase inhibitors are reserved exclusively for use during or immediately before a pandemic; they are not intended for day-to-day use on the same scale. In contrast, antimicrobial drugs are widely used every day. This difference means that antimicrobial drugs could act as buffer stock (conceptually similar to vendor-managed inventory) in most healthcare systems, rather than a true stockpile. Indeed, the word *stockpile* may be a misnomer in relation to increased stores of antimicrobial drugs because these drugs can be channeled into day-to-day use and replaced through fresh procurement. Thus, over time the amount, proportion, and range of these agents held can be slowly altered. These 2 mechanisms, ongoing interpandemic use and restocking, make such a stockpile far less vulnerable than antiviral drugs to expiration before use and far more responsive to changes in antimicrobial drug sensitivity detected between the date of procurement and the onset of the next pandemic.

Further considerations relate to storage. Whereas antiviral drugs and vaccines essentially need to be held in secure centralized storage (the latter within the cold chain) until eventual deployment, antimicrobial drugs can be held, at least in part, lower down the supply chain by wholesalers and community pharmacies or their equivalent.

Additionally, the proportion of pandemic influenza cases that will progress to bacterial complications needs to be estimated. The difficulty in making such an estimate relates partly to the paucity of contemporary data that specifically describe the incidence of bacterial complications after influenza and partly to the fact that widespread use of neuraminidase inhibitors, rarely used for seasonal influenza, might reduce the development of antimicrobial drug–related complications by 25%–40% (*24*,*25*). Data from the extensive reviews by Brundage and Soper suggest that in the 3 pandemics of the 20th century, bacterial pneumonia developed in 15%–20% of influenza patients (*2*,*26*); some estimates for seasonal influenza are far higher (*27*). It can be argued that in 1918 the primary viral infection was so virulent that it caused the premature demise of some patients who might otherwise have survived long enough for bacterial pneumonia to develop; i.e., the reported frequency of bacterial complications was spuriously low. Coupled with a population clinical attack rate that will most likely lie in the range of 25% to 50%, an antimicrobial drug stockpile is likely to be needed for a minimum of 10% of the population. This figure does not account for wastage, misdiagnosis (if, as is most likely, prescribing is based on clinical suspicion alone), or a higher rate of secondary bacterial complication than expected; it is also based on a strategy of treatment only.

### Treatment and Prophylaxis Strategies

Alternative strategies include offering antimicrobial drug prophylaxis at the same time as antiviral treatment to patients with conditions that put them at high risk, such as chronic obstructive pulmonary disease. Antimicrobial drugs (or a prescription for them) could be issued to high-risk patients at the same time as antiviral treatment. The ability to start antimicrobial drug therapy with minimal delay and without the need for repeat consultation if antiviral drugs alone are not effective might be advantageous in an already overstretched health system. Both of these strategies incorporate further uncertainty because the high-risk groups in a pandemic are unknown and may not correspond to those currently recognizable for seasonal influenza; if anything, the high-risk groups are more likely than not to be larger in a pandemic. This might increase the requirement for antimicrobial drug stockpiling to 25% population coverage.

Each country should estimate its own needs. Country-specific factors to take into account include treatment strategy (treatment alone or treatment and prophylaxis), health service configuration, historical use of antimicrobial drugs, physician behavior, inappropriate prescribing linked to misdiagnosis, and the availability of antiviral drugs.

### Quinolone Stockpiles

A large number of countries hold stockpiles of quinolones, in particular ciprofloxacin, as a contingency against bioterrorist threats. In the United Kingdom, ciprofloxacin is active against all *H. influenzae* isolates (≈100% of recent UK respiratory tract isolates susceptible; HPA, unpub. data), most methicillin-sensitive *S. aureus* isolates (≈82%), and atypical organisms. Therefore, if these bacterial pathogens were known or suspected to predominate in influenza-related pneumonia associated with a future pandemic, the use of ciprofloxacin might be justified, and agents effective against MRSA would be reserved for severe cases and those with culture-confirmed MRSA (99% of UK respiratory MRSA isolates, most of which are hospital acquired, are quinolone resistant; HPA, unpub. data).

However, ciprofloxacin activity against *S. pneumoniae* (*28*) is only intermediate, and a significant number of bacterial pneumonias complicating influenza may not respond to empirical treatment. This fact is well supported by evidence from mouse models; more modern “respiratory” fluoroquinolones such as gatifloxacin demonstrate good results (*29*) against *S. pneumoniae,* which was not always so for ciprofloxacin. Therefore, in a pandemic empirical ciprofloxacin use could be justified only if all other more suitable antimicrobial drug supplies were exhausted.

Given ciprofloxacin’s weak activity against pneumococci, reserving its use in a pandemic to empirical treatment of persons previously vaccinated against pneumococcal infection, who would be at reduced risk for co-infection with this particular organism, would be reasonable. Theoretical support for this hypothesis comes from the United States, where use of a 7-valent conjugate vaccine since 2000 has resulted in declining invasive pneumococcal disease (*30*) and relatively infrequent influenza-related deaths caused by pneumococci in children (*5*). A strategic approach might involve the use of ciprofloxacin in fully immunized persons.

### Pneumococcal Vaccination Strategies

Including a vaccination strategy in pandemic planning would potentially reduce the amount of disease caused by secondary *S. pneumoniae* bacterial pneumonia. We have already described this pathogen’s role in community-acquired pneumonia and influenza complications. The public health benefit from vaccination could be substantial.

Pneumococcal polysaccharide vaccine (PPV) is currently recommended in many countries for persons >65 years of age and for high-risk groups of all ages. Few specific data exist on the effectiveness of PPV for reducing pneumococcal pneumonia–associated illness and death after infection with influenza A virus. Furthermore, in the context of pneumococcal disease not specifically associated with influenza, use of PPV has protected against invasive pneumococcal disease but not against pneumococcal pneumonia in the absence of bacteremia (*31*). Therefore, on the basis of current evidence, prior PPV administration could not reliably be used to identify persons who could receive empirical ciprofloxacin therapy for bacterial pneumonia as a complication of influenza. It could, however, be used as a large-scale preventive measure against invasive pneumococcal disease in adults.

The protective efficacy of a 9-valent pneumococcal conjugate vaccine (PncCV) against nonbacteremic pneumonia as well as invasive pneumococcal disease has been demonstrated in 37,107 children from South Africa among whom the prevalence of HIV infection was 6.5% (*32*).The vaccine also substantially reduced the incidence of first episodes of invasive pneumococcal disease that were resistant to penicillin or trimethoprim-sulfamethoxazole.

PncCV may have more greatly reduced the incidence of pneumonia in children when a virus was isolated (*33*). This effect was more pronounced when influenza A was isolated; protective efficacy was 41% (95% confidence interval 13%–60%). The study provided indirect evidence of the frequency of pneumococcal superinfection of viral pneumonias in children in this setting. If similar results could be achieved through vaccination before an influenza pandemic, the benefits of preventing pneumococcal complications could be substantial. The introduction of conjugate vaccine in the United States in 2000 has led to a decline in invasive pneumococcal disease in not only children but also adults; reduction was 32% for those 20–39 years of age and 18% for those >65 years (*30*). Therefore, vaccination of children might be the most cost-effective policy. In September 2006, the United Kingdom started vaccinating children from the age of 2 months; early unpublished data (minutes from the Joint Committee on Vaccination and Immunisation meeting on February 14, 2007, available from www.advisorybodies.doh.gov.uk/jcvi/mins140207.htm) suggest that invasive pneumococcal disease in children <2 years of age is already reduced.

The use of PncCV in children and 23-valent PPV in adults as part of a pandemic strategy would be consistent with recommendations resulting from current published data. However, such use may still not allow for ciprofloxacin stockpiles to be reliably targeted for specific populations, given the lack of protection against nonbacteremic pneumoccal pneumonia associated with PPV. If conjugate vaccine were used in all patients (although no convincing data exist to support efficacy of conjugate vaccine in adults), ciprofloxacin might be more reliably targeted at a group more likely to have a nonpneumococcal pneumonia. However, a conjugate vaccine is likely to be expensive and limited in serotype coverage, and approval for its use in adults will take time.

## Discussion

Substantial laboratory and epidemiologic evidence shows that influenza A and bacterial pathogens often participate in the pathogenesis of pneumonia. Several issues need to be considered with regard to antimicrobial drug treatment for large numbers of patients who have secondary bacterial infection during a pandemic. Real-time antimicrobial drug–resistance surveillance programs could be incorporated into preparedness frameworks; information from such networks could result in stockpiling of inexpensive, generically manufactured antimicrobial drugs. Vaccination against pneumococcal disease, particularly vaccination of HIV-infected persons, potentially will save lives in the short term as well as provide protection in the event of a pandemic.
